# Developing a conversion rate optimization framework for digital retailers—case study

**DOI:** 10.1057/s41270-022-00161-y

**Published:** 2022-02-23

**Authors:** Robert Zimmermann, Andreas Auinger

**Affiliations:** Digital Business Management, University of Applied Sciences Upper Austria, Wehrgrabengasse 1-3, 4400 Steyr, Austria

**Keywords:** Digital retail, Framework, Conversion rate optimization, Case study

## Abstract

To stay competitive against e-commerce, many retailers started to adopt a digital retail strategy, leveraging a myriad of online and offline touchpoints to increase their customer experience and, as a result, their sales. However, currently, no guidelines exist on how digital retailers can identify, evaluate, and influence sales impacting touchpoints along the customer journey. Hence, this study derives key elements of a conversion rate optimization framework, which can be used to increase sales of a digital retailer. Additionally, the derived framework is tested with the Austrian subsidiary of an international sports appeal and equipment retailer giving insights into its practical applicability. Results indicate that the developed framework can indeed be used to identify sales influencing touchpoints, which can be altered by specific marketing actions to increase sales of a digital retailer.

## Introduction

Traditional retail is facing significant challenges including the following: department store revenue peaked in 2001 and has been declining ever since (ICSC 2018; US Census Bureau 2018), e-commerce revenue is constantly growing (Statista 2021), and COVID-19 social distancing measures reduced footfall to a minimum (Retail Gazette 2021) leading to the bankruptcy of 15,542 department stores in 2020 in the US alone (Loeb 2020).

Responding to these challenges, retailers started to adopt digital retail strategies, which, in this study, are defined as the empowerment of physical retailing businesses through digital technology which allows retailers to operate new channels and customer contact points (hereafter: touchpoints). As such, digital retailers use information and communication technologies (such as smartphones) to attract customers, drive sales, and provide a unique customer experience that is superior to the more traditional online customer experience (Lemon and Verhoef 2016). Making use of these technologies, retailers quickly began to target clients across various channels separately, with each channel being treated as disconnected from the others (Anderl et al. 2016), bringing forth what is presently known as the multi-channel retail approach. However, as stated by Melero et al. (2016) and Parise et al. (2016), treating channels separately often leads to a subpar customer experience, which is why multi-channel retail is progressively changing into omnichannel retail, as it integrates all channels and touchpoints into a single seamless and enhanced consumer experience (Verhoef et al. 2015).

Although this omnichannel experience can help digital retailers increase their competitive power it also presents additional challenges. Especially, the ever-increasing number of touchpoints customers can encounter along the omnichannel customer journey which contains online as well as offline touchpoints, proves to be problematic, as the ability of a digital retailer to steer and manage an ever-growing customer journey is limited (Lemon and Verhoef 2016; Rosenbaum et al. 2017). Additionally, digital retailers struggle to assign specific values to touchpoints as their perceived value or even the number of recognized touchpoints can differ significantly between customer and company (Rosenbaum et al. 2017) and even inside companies themselves (Zimmermann and Auinger 2021). This is especially problematic as digital retailers do not know the precise impact on sales of a single touchpoint along their customer journey.

It becomes evident that retailers need to make the most out of the dwindling number of customers they can interact with along their ever-increasing customer journey, or, in other words, retailers require a conversion rate optimization framework, whose usage leads to the identification of the most sales influencing customer touchpoints and specific actions how to influence them. Therefore, the overall goal of this paper is to: “Offer possible solutions to the set-out issues of digital retail and to use these solutions as building blocks in a conversion rate optimization framework”.

It must be noted that although many touchpoints are designed to foster other types of conversions besides sales (e.g., email signups, shares on social media), this study focuses on the sales influencing effect of touchpoints and understands conversion rate optimization as the process of generating additional sales by first identifying the most sales influencing touchpoints of a digital retailer and second, offering appropriate marketing actions to use these touchpoints in a way which increases sales the most.

Thus, this study illuminates the presented issues of digital retail and offers possible solutions in Chapter 2. Following on from this, Chapter 3 condenses the identified solutions into a conversion rate optimization framework, which is then exemplified and has its results discussed in a case study of an Austrian subsidiary of an international sports appeal and equipment retailer presented in Chapter 4. Based on the results of the case study, possible managerial and scientific contributions of the framework are discussed in Chapter 5. Additionally, the paper depicts possible limitations of the proposed framework, future research opportunities, and a conclusion in Chapter 6.

## Issues of digital retail and possible solutions

As pointed out in the introduction, digital retail, besides having the potential to increase customer experience and sales, also presents additional challenges. Reflecting on a key article (3297 citations based on Google Scholar on the 07.02.2022), outlining the biggest problems in customer experience management throughout the customer journey written by Lemon and Verhoef (2016), we derive the following issues digital retailers will have to solve in order to make use of sales increasing customer experiences. Namely, the ever-increasing customer journey, the differing value perceptions of touchpoints, and the inability to determine the impact of specific touchpoints on sales. In this chapter, these issues are illuminated together with possible solutions.

### The ever-increasing customer journey

Through the combination of online and offline customer journeys in digital retail the number of touchpoints customers can encounter is constantly increasing (Lemon and Verhoef 2016) and as pointed out by Rosenbaum et al. (2017) drawing out a customer journey map including all existing touchpoints would result in an overly complex overview with little management value. However, Lemon and Verhoef (2016) point out that although being a difficult task, the identification of the most influential touchpoints of a retailer is crucial if a retailer wants to influence customer decisions along the customer journey. Therefore, they argue that retailers require at least a comprehensive overview of all brand-owned touchpoints of a digital retailer. Brand-owned touchpoints hereby represent touchpoints that are designed and managed by the retailer only (e.g., advertising, corporate websites, owned social media channels, in-store displays). To give an example, a video hosted by a retailer on the platform YouTube.com is considered a brand-owned touchpoint as the retailer is in complete control of the video (content, upload time, etc.). In contrast, a video uploaded by a customer on the same platform about the retailer or one of its products would not be considered a brand-owned touchpoint, if the customer did not receive any incentive from the retailer to upload the video, as the retailer cannot control a single aspect of the video.

Regardless, even identifying brand-owned touchpoints only still represents a challenging task. As illustrated by Berendes et al. (2018), the most well-known customer journey mapping techniques (Bitner et al. 2008; Haugstveit et al. 2016; Patrício et al. 2011; Rosenbaum et al. 2017; Teixeira et al. 2012), derive from a service innovation or service delivery background and thus try to model one specific customer journey of one specific group of customers to improve specific service quality and service delivery. Hence, none of these techniques has the intention to create an all-inclusive overview of brand-owned touchpoints. However, a first attempt in designing an all-inclusive overview of brand-owned touchpoints was demonstrated by Zimmermann and Auinger (2020). They developed a workshop, which used the creative techniques, World Café (Brown and Isaacs 2007) and Channel CARDS (foryouandyourcustomers.com 2022), to extract knowledge about brand-owned touchpoints from employees until they reach a state of data saturation in which now additional knowledge can be extracted from said employees. Thus, it can be concluded that a possible solution to the ever-increasing customer journey of digital retailers is to focus on brand-owned touchpoints and to acquire a comprehensive overview of at least all brand-owned touchpoints as they represent the only touchpoints a digital retailer can reliably influence.

### The value perception of touchpoints

Lemon and Verhoef (2016) point out that touchpoints can create strong, positive experiences within the customer journey and thus lead to an increase of, for example, the sales conversion rate. However, the perceived value of touchpoints can differ significantly between a company and its customers (Rosenbaum et al. 2017), in the sense of which touchpoints are actually recognized by customers, as well as inside a company (Zimmermann and Auinger 2021), in the sense of differing perceptions about the importance of touchpoints for the company. This leads to multiple problems. Evidently, if a specific touchpoint is highly valued by a company, but is not even recognized by its customers, the actual value generated by such a touchpoint might be much lower than anticipated and vice versa. Therefore, it is crucial to identify the touchpoints, which according to customers, are most important on their customer journey. As stated by Grewal and Roggeveen (2020), it is of vital importance that these touchpoints are designed and integrated optimally into the digital retailer’s customer journey. However, how managers rate the importance of a touchpoint has an impact on how they handle them (Liu and McMurray 2004) and as shown by Zimmermann and Auinger (2021) the perceived importance inside a company can differ significantly between managerial and departmental levels. This implies that digital retail activities can lack efficiency and effectiveness as even on a company level no unique value perception of touchpoints is present, which can easily lead to a mismatch in company resource alignment, prioritization, and thus goal fulfillment. A possible solution to reveal differences in value perception could be customer and employee surveys evaluating the exact value perception of at least all brand-owned touchpoints (Zimmermann et al. 2022; Zimmermann and Auinger 2021). This would allow digital retailers to streamline the companywide customer journey management strategy making it more effective and consistent. Additionally, it would enable them to either focus their business efforts on touchpoints recognized by customers or to light up blind spots along the customer journey that customers do not yet recognize and thus do not perceive as valuable.

### The impact of a touchpoint on sales

One of the main reasons to follow a digital retail strategy is to increase customer experience and consequently increase sales (Bradley et al. 2015; Juaneda-Ayensa et al. 2016; Parise et al. 2016). However, determining the impact of a single touchpoint on sales has always been a difficult endeavor. Thus, various marketing attribution models (e.g., First Interaction, Last Interaction, Linear, Time-Decay) have been developed, which try to distribute the value of a sale across the customer journey (AgencyAnalytics 2020). However, as stated by Lemon and Verhoef (2016) attribution models do not adequately account for the constant channel switching during the nonlinear digital retail customer journey, which does not necessarily have a fixed start or end. Trying to account for nonlinearity, various statistical models have been developed to attribute sales across online customer journeys (Li and Kannan 2014; Xu et al. 2014). However, these are also not transferable to digital retail as they exclude the offline part of the digital retail customer journey, on which customer interactions are especially difficult to track (Lemon and Verhoef 2016). Offering a possible solution to these problems, Zimmermann et al. (2022) combined a statistical approach with a customer survey and sales data to analyze the direct effect of individual online and offline touchpoints on the money generated by a customer encountering these touchpoints.

## Digital retail conversion rate optimization framework

**Fig. 1:**
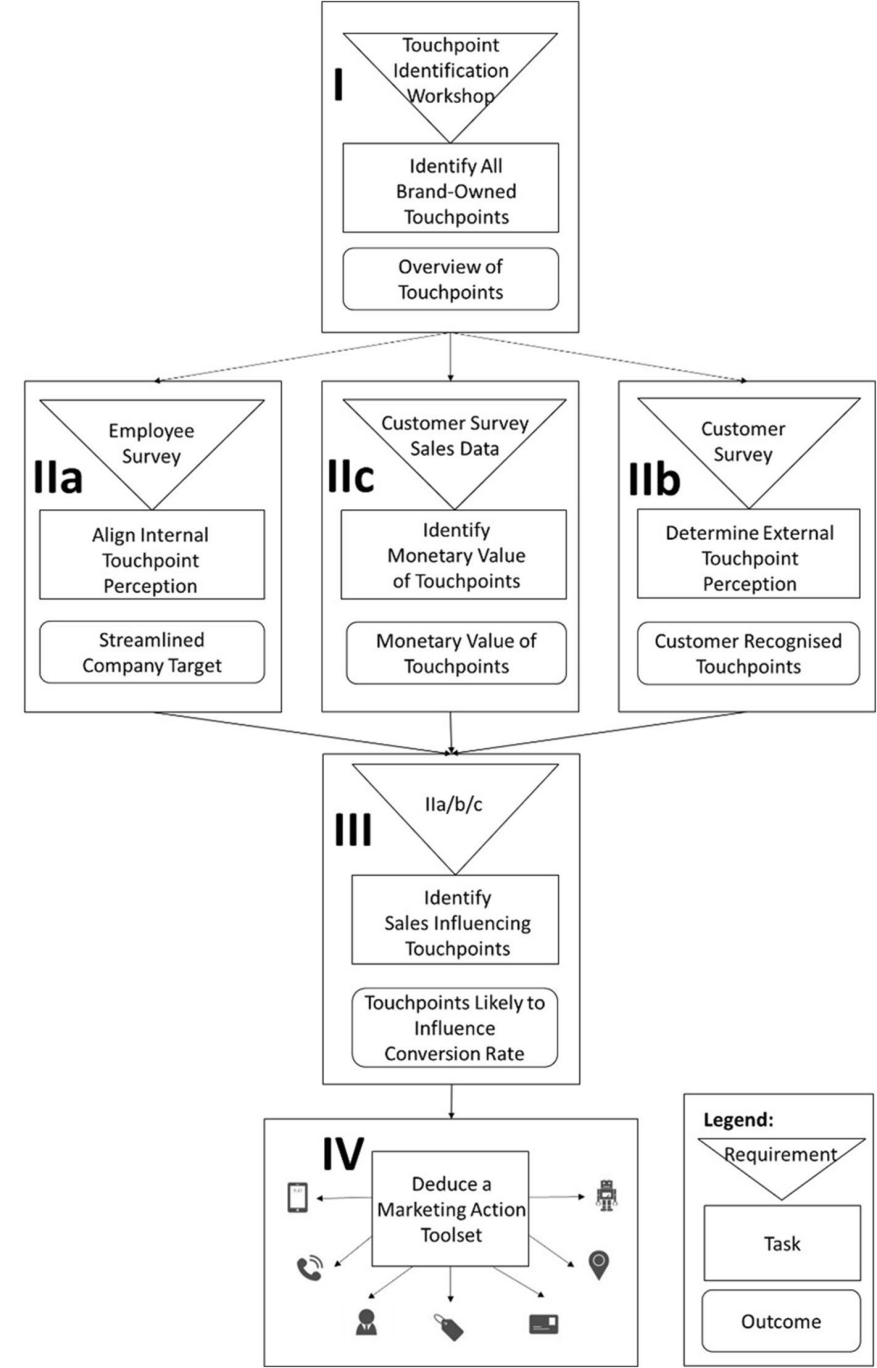
Conversion rate optimization framework

To the best knowledge of the authors, currently, no conversion rate optimization framework for digital retailers exists. Although previous studies attempted to map the customer journey (Bitner et al. 2008; Haugstveit et al. 2016; Patrício et al. 2011; Rosenbaum et al. 2017), determine the impact of website touchpoints on sales (Li and Kannan 2014; Xu et al. 2014), and indeed multiple conversion rate optimization frameworks for websites do exist (Goward 2009; Moogan 2014; Pritesh 2020; Saleh 2017), no single piece of work has tried to build a conversion rate optimization framework, which considers the duality of online and offline touchpoints along the digital retail customer journey. Therefore, this paper uses the presented solutions from the previous chapter as building blocks for a conversion rate optimization framework, which is specifically tailored to the requirements of digital retailers. The overall framework is shown in Fig. 1. It describes the steps required to generate a marketing action toolset, which accumulates the most appropriate marketing actions a digital retailer can use to influence touchpoints, which have a high probability to increase conversion rate.

### Step I: Identify all brand-owned touchpoints

Knowing which influenceable touchpoints a digital retailer offers its customers is crucial if a digital retailer wants to influence any decision of a customer. To acquire a comprehensive overview of brand-owned touchpoints, a “Touchpoint Identification Workshop” [e.g., as described by Zimmermann and Auinger (2020)] should be performed. Such a workshop should include people from all departments and all managerial levels, to ensure that a companywide knowledge base is used to generate a data saturated overview of brand-owned touchpoints. The resulting “Overview of Touchpoints” forms the basis for all following steps.

### Step II: Align internal & determine external touchpoint perception

**IIa** Using the gained overview of touchpoints together with an “Employee Survey”, digital retailers should “Align their Internal Touchpoint Perception”. In this regard, the employee survey should evaluate how different departments and managerial levels perceive the value of the previously identified brand-owned touchpoints. As a result, retailers can identify and even out differing perceptions about the importance of touchpoints inside the company, which, according to Grewal and Roggeveen (2020), are key requirements to create a streamlined companywide customer journey management strategy. It has to be noted that the definition of what constitutes value has to be done by each digital retailer individually.

**IIb** Additionally, the overview of touchpoints should be used in a “Customer Survey” to “Determine the External Touchpoint Perception”. This survey should evaluate which brand-owned touchpoints are actually recognized by customers and thus allows digital retailers to either focus their business efforts on touchpoints recognized by customers or light up blind spots along the customer journey that customers do not yet recognize as anticipated by the digital retailer.

**IIc** Following on from this, “Sales Data” and a “Customer Survey” should be combined with statistical analysis to “Determine the Monetary Value of Touchpoints”. In contrast to the previous steps, (IIa, IIb) which generated soft data, this step should generate hard data to offer an additional perspective for upcoming discussions about the influence of specific brand-owned touchpoints on sales (III).

### Step III: Identifying sales influencing touchpoints

Using the knowledge about the monetary impact of touchpoints (IIc), together with the understanding of the streamlined company targets (IIa) and realizing which touchpoints are recognized and thus valued by customers (IIb), allows digital retailers to identify touchpoints that are most likely to influence conversion rate. This is because digital retailers now know which touchpoints present the highest value for them from a company, a customer, and a statistical perspective.

### Step IV: Derive a marketing action toolset for conversion rate optimization

Having completed the previous steps, the digital retailer should now be well informed about the impact of the retailer’s brand-owned touchpoints on sales. However, this knowledge must still be converted into a sales increase. Therefore, a digital retailer must now intensely discuss why a specific touchpoint does have an influence on sales and if it is a touchpoint worth tinkering with. If the digital retailer can figure out the specific reasons why a touchpoint has an influence on sales and concludes it can be influenced with a reasonable amount of effort, they can derive specific marketing actions to influence this specific touchpoint. As this touchpoint has previously been identified as having an influence on sales by the performed statistical analysis (IIc), is highly valued by customers as seen in the customer survey (IIb), and is part of the touchpoints the digital retailer wants to present on the streamlined customer journey as identified in the employee survey (IIa), we argue, there is a high probability that influencing such touchpoints with appropriate marketing actions will lead to an increase in conversion rate.

## Case study

Following Yin (2003), we conducted a descriptive single case study with embedded units presenting an actual use case of the framework, which was tested with the Austrian subsidiary (> 250 stores, > 3500 employees, > 550€ million turnover) of an international sports appeal and equipment retailer (> 11.5€ billion turnover) which recently adopted a digital retail strategy. This chapter demonstrates how the digital retailer performed the individual steps proposed in the framework and, which results were gained from completing each step.

### Case study Step I: identify all brand-owned touchpoints

To identify all brand-owned touchpoints the retailer used the workshop design proposed by Zimmermann and Auinger (2020). As such, a selected group of persons, which represented all major departments and managerial levels of the retailer, were invited to a one-day workshop. After being instructed, the employees performed a world café and started to gather touchpoints for four specific personas previously identified by the retailer.

Following, the group of employees was divided into two focus groups which started to identify touchpoints with the help of the creative technique channel cards. The results of the two groups were compared using the statistical measure of Cohen’s Kappa. As the Kappa value reached 0.76, a substantial agreement (Landis and Koch 1977) and thus a state of data saturation was reached.

The two groups joined back together and discussed how to cluster the identified touchpoints into groups with the help of instant polls and instant word clouds. This resulted in a data saturated overview of 145 brand-owned touchpoints clustered into 13 categories. However, after the workshop, the top management of the retailer re-evaluated the overview and decided to integrate one of the 13 categories into another (the reason being the redundancy of the category) leaving a total of 12 categories (“POS”, “Website”, “Service”, “Print”, “Online Advertisement”, “Social Media”, “Cooperations”, “Customer Relationship Management”, “Public Relations”, “Classic Media”, “Out of Home”, “Sponsoring”, “Events”).

### Case study Step II: align internal & determine external touchpoint perception

#### IIa align internal touchpoint perception

The gained overview of brand-owned touchpoints was used in a subsequent survey, which was sent to all employees of the Austrian subsidary’s headquarters. The survey presented the identified brand-owned touchpoint from each category to the employees, asking how the employees, taking a customers’ perspective, would rate the influence of the respective touchpoint and category on brand perception on a 5-point Likert scale (range: 1 = ‘Has no influence’ to 5 = ‘Has extreme influence’). Looking at the management levels, the employee’s response rate on average was 30%. However, as the response rates of the higher managerial levels (C-level, Head of department, Team leader) were high, the sample was regarded as sufficient to show differences in the perceived influence of brand-owned touchpoints on brand perceptions across the different managerial levels and thus as representative. The response rate from the company’s departments on average was 33%. However, it must be noted that every department with less than five survey participants, except for the management board, was excluded from the analysis. As five out of ten departments had a response rate of over 40%, the sample was regarded as representative to show differences in the perceived influence of brand-owned touchpoints on brand perceptions across the differing departmental levels.

Looking at the mean differences between the perceived influence of brand-owned touchpoints on the brand perception of the C-level compared to the other management levels revealed differences ranging between plus 33% (Head of department/“Events”/absolute difference 1.0) and − 20% (Executive/“CRM”/absolute difference − 0.89). On the departmental level, the mean differences in the perceived influence of brand-owned touchpoints on brand perception between the management board and the other departments ranged from plus 39% (Marketing/“Print”/absolute difference 1.17) to − 40% (Product Management/“Social Media”/absolute difference − 1.87). As the analyses are based on representative samples, it was concluded that these observed differences are indeed currently present between the managerial and departmental levels of the retailer although not always statistically significant.

Thus, the analysis revealed that the company’s highest managerial and departmental authorities (C-level/Management board) were not able to communicate their exact view of the company’s brand-owned touchpoints to the lower levels of management or other departments. From a managerial point of view, this is problematic as Grewal and Roggeveen (2020) state today’s digital retailers need a systematic and integrated customer journey management to optimize product placement, service, and communication. Therefore, the top management of the retailer argued that they need to harmonize the managerial as well as departmental levels of their company. On the managerial level, they concluded that differences in the perceived influence of brand-owned touchpoints on brand perception should be discussed, understood, and ultimately evened out to ensure a consistent companywide customer journey management strategy. On the departmental level, top management concluded that the differences in the perceived influence of brand-owned touchpoints should also be discussed and understood but ultimately utilized, allowing the different departments to make use of their differing perceptions of brand-owned touchpoints and thus foster creative and innovative handling of brand-owned touchpoints in the future.

#### IIb Align external touchpoint perception

The identified brand-owned touchpoints were also used in a subsequent online survey to identify the most recognized touchpoints from a customer perspective. In this online survey, participants were asked when they brought a product from the retailer the last time (options: ‘In the past 3/6/9/12 months’, ‘More than 12 months ago’, ‘Never’). Following, they were presented with the 12 previously identified main touchpoint categories and were asked to rate how often they came into contact with the touchpoint categories. This was measured on a 5-point Likert scale (range: 1 = ‘Not at all’ to 5 = ‘Very often’). For every main category in which participants did not answer ‘Not at all’, they were shown a follow-up question presenting all touchpoints of this main category. Consequently, participants were asked to rate how often they recognized specific touchpoints on a 5-point Likert scale (range: 1 = ‘Not at all’ to 5 = ‘Very often’). The retailer distributed the survey to 76,948 registered customers of the retailer between mid-November and mid-December 2020. Of the participants, 1487 successfully completed the survey. From these, only questionnaires of those individuals for whom the retailer was able to provide a detailed sales history, including online and offline sales, were analyzed. Additionally, as suggested in the literature (Leva and Ziliani 2018), the retailer only selected customers who had made a purchase within the last three months to keep recall bias to a minimum and to ensure that the customers’ memory of used touchpoints was reliable. Lastly, the sample was cleaned of outliers and participants whose answers were incomplete. *N* = 243 participants remained, who were further analyzed.

An exploratory factor analysis was used to extract the underlying structure of the identified 145 touchpoints (i.e., touchpoint categories) from the customer perspective. The Bartlett-Test (*χ*^2^ = 20,293.879, df = 6441, *p* < 0.001) as well as the Kaiser–Meyer–Olkin Measure of Sampling Adequacy (KMO = 0.837) indicated that the identified touchpoints are suitable for factor analysis. Hence, a principal component analysis with varimax rotation was performed. Based on the scree plot and theoretical considerations, the retailer chose a 7-factor solution explaining 46.04% of the variance. The extracted factors (i.e., touchpoint clusters) were: ‘Point of Sale’, ‘Cooperations’, ‘Service’, ‘Website’, ‘Classic Media’, ‘Social Media’, and ‘Customer Relationship Management’. Moving forward, the retailer used confirmatory factor analysis to reassess the identified structure. The model fitted the data adequately (*χ*^2^ = 794.5, df = 443, RMSEA = 0.057, CFI = 0.911, SRMR = 0.059) (Coughlan et al. 2008; Little and Kline 2016). Construct validity and reliability were also established as indicated by adequate Cronbach’s alpha coefficients (range 0.753–0.924), sufficient item to construct loadings (range 0.516–0.899), and composite reliability (range 0.757–0.886). The average variance extracted (AVE) ranged between 0.539 and 0.668 and was above the desired threshold of 0.5. Only the factors ‘Cooperation’ (0.421) and ‘Classic Media’ (0.338) were below. However, as stated by Fornell and Larcker (1981), an AVE of less than 0.5 can still be regarded as adequate if composite reliability is above 0.6, which was true for all factors. Discriminate validity was also established as the square root of all AVE values exceeded the corresponding correlations for all factor pairs (Fornell and Larcker 1981).

Results indicate that the seven touchpoint clusters most recognized by customers are: ‘Point of Sale’, ‘Cooperation’, ‘Service’, ‘Website’, ‘Classic Media’, ‘Social Media’ and, ‘Customer Relationship Management’. In the initial touchpoint identification workshop conducted with the retailer (Step: I), however, twelve main categories were identified by the retailer’s employees (including top managers). This means that the retailer follows a different logic than its customers do when organizing the brand-owned touchpoints. However, especially the way managers group touchpoints has an impact on how they coordinate them (e.g., communicating a unified marketing message within a specific category) (Liu and McMurray 2004; Zimmermann and Auinger 2021). This implies that the digital retail activities of the retailer lack efficiency and effectiveness since the same marketing messages can be communicated in a seemingly homogeneous group of touchpoints, while customers typically tend to use touchpoints that (according to the retail managers) belong to different touchpoint clusters. For the retailer analyzed, the customer-based clusters represent the most important points of interest the retailer can use to interact with customers in a consistent way. Therefore, in line with Grewal and Roggeveen (2020), the retailer decided to especially care for their optimal design and integration into the retailer’s customer journey. For touchpoints, in which the retailer put a lot of effort, and which were not part of a customer-based cluster, the retailer readjusted the promotion strategy of these touchpoints, as it seems not to be recognized by customers yet. In summary, the retailer could identify potential gaps between the managers’ and customers’ viewpoints concerning touchpoints recognition and usage.

#### IIc Determine the monetary value of touchpoints

Using the data from the survey executed in IIb (*N* = 243), the retailer performed a Bayesian regression with the touchpoint recognition of the 145 identified touchpoints (measured on a 5-point Likert scale, range: 1 = ‘Not at all’ to 5 = ‘Very often’) as independent and the sales value generated (money spent by a customer) as dependent variable. As prior for the regression coefficients the retailer used the Jeffreys-Zellner-Siow prior with an *r* scale of 0.354, as this is the most recommended prior in literature (Andraszewicz et al. 2015; Heo and van de Schoot 2020). Prior model probability was set to uniform as the retailer regarded all touchpoints as equally likely to influence the sales value of a customer and this option presents the most neutral choice (Hoeting et al. 1999).

Results of the Bayesian regression showed multiple models were more likely to predict the direct influence of specific touchpoints on sales than a Null model, which states that the independent variables do not predict the dependent variable. More specifically, the best-calculated model was 10,739 times more likely to predict the dependent variable (No: 1, *R*^2^ = 0.162, BF10 = 10,739) compared to the Null model. According to Lee and Wagenmakers (2014), this presents extreme evidence that the independent variables can predict the dependent variable.

Additionally, the Bayesian regression was able to identify a set of touchpoints that were most likely to influence the sales value of a customer from a purely statistical point of view. For example, the touchpoint ‘Warranty services’ showed that an increase of one Likert point in warranty service recognition increased the sales value of a customer, on average and across all models, by €47.40. Every model containing ‘Warranty service’ as a predictor is 403.94 times more likely to predict sales value (BF inclusion = 403.94). Contrary, an increase in recognition of the touchpoint ‘Digital signage outdoor’ worth one Likert point, will decrease the average sales value of a customer by €36.95, meaning that when a customer recognizes this touchpoint, it has a negative impact on their expenditure. Every model containing ‘Digital signage outdoor’ as a predictor was 24.194 times more likely to predict sales value (BF inclusion = 24.194).

To summarize, the Bayesian regression was able to identify specific touchpoints that influence the sales value of a customer.

### Case study Step III: identifying sales influencing touchpoints

From the retailer’s perspective, all these effects must be put into context with the previously identified streamlined company targets (IIa Internal Touchpoint Perception), the touchpoints actually recognized (i.e., valued) by customers (IIb External Touchpoint Perception), and the monetary value of touchpoints determined by the statistical analyses (IIc Determine the Monetary Value of Touchpoints), to identify sales influencing touchpoints.

Taking the touchpoint ‘Warranty Service’ as an example. The touchpoint was recognized by the retailer and its customers as an important touchpoint and according to the Bayesian regression, has a positive impact on sales. It thus represents a sales influencing touchpoint for the retailer. Evaluating the influence of the touchpoint, the retailer argued that customers with a high sales value often make use of the warranty service, which could indicate high product returns because of poor product quality. Another developed interpretation was that the touchpoint ‘Warranty Service’ could generate a feeling of security in customers who buy many products because of the availability of warranty services in general. Another example of this process to identify sales influencing touchpoints was the touchpoint ‘Cooperations’. This touchpoint was also recognized by the retailer and its customers as an important touchpoint, however, according to the Bayesian regression, has a negative impact on sales. Thus, it also represents a sales influencing touchpoint for the retailer. The retailer argued that the negative influence of cooperations should not result in the abandonment of all retail cooperations. Instead, the retailer decided to evaluate why this factor reduces customer sales value and what can be done to improve the perception of this factor.

It became evident that, in general, touchpoints that impose opinions on customers (mostly marketing communication channels) had a negative impact on sales value, while service channels had a positive impact for the retailer. Following Gatignon (2016), a plausible explanation would be that customers do not like to be manipulated, but enjoy the services provided by the retailer.

### Case study Step IV: derive a marketing action toolset for conversion rate optimization

Having identified sales influencing touchpoints, the retailer started to derive specific marketing actions to influence these touchpoints. In the following, two of these marketing actions are exemplified with the touchpoints ‘Warranty Service’ and ‘Cooperations’.

As argued by the retailer, the touchpoint ‘Warranty service’ could affect sales value either by poor product quality or by satisfying the customers' need for security by having a warranty service in place. Addressing the product quality itself with marketing actions is not possible. However, the retailer could work on their product description or price to align the expectations of the customers with the true product quality to reduce customers’ dissatisfaction. In contrast, highlighting the warranty services through various marketing channels (newsletter, social media, sales personnel, etc.) could increase the positive perception of the touchpoint even more, and thus have an additional positive effect on sales.

The retailer argued that the touchpoint ‘Cooperations’ has a negative effect on sales value but did want to abandon cooperations but instead revaluate them. After readjusting targeting, messages delivered, and cooperation partners themselves, a derivable marketing action could be to use the “right” marketing channels to deliver the “right” cooperation to the “right” customer using personalized content marketing actions.

As exemplified with the presented examples, the number of potential marketing actions is limited only by the creativity of the marketing department of the retailer. However, after every marketing action, an evaluation of the impact of the performed actions on sales should be carried out to ensure that developed marketing actions indeed have a positive effect on the conversion rate of the digital retailer.

## Discussion

Reflecting on the results of the case study, managerial as well as scientific implications can be discussed.

Regarding the “Ever-increasing Customer Journey”, the case study demonstrated that the workshop design proposed by Zimmermann and Auinger (2020), indeed represented a feasible way for the digital retailer to identify all of its brand-owned touchpoints as the workshop could reach a state of data saturation (*κ* = 0.76). Thus, we argue that this workshop design should especially be used in circumstances when modellng single customer journeys, as proposed by traditional customer journey mapping techniques [e.g., (Bitner et al. 2008; Haugstveit et al. 2016; Patrício et al. 2011; Rosenbaum et al. 2017; Teixeira et al. 2012)], is not sufficient and a general overview of all brand-owned touchpoints is required.

Investigating the “Value Perception of Touchpoints”, the case study exemplified that using an employee survey the internal touchpoint perception, and using a customer survey, the external touchpoint perception could be determined by the digital retailer. Hereby, differences in the perceived influence of brand-owned touchpoints on brand perceptions were present on all managerial and departmental levels. Additionally, it could be demonstrated that customers recognize a different set of touchpoints than the retailer anticipated. Grewal and Roggeveen (2020) argue that an optimal design and integration of touchpoints is crucial to ensure effective customer journey management. Thus, we argue that the framework allows digital retailers to streamline the companywide customer journey management strategy making it more effective and consistent. This allows them to focus their business efforts on touchpoints recognized by customers and to light up blind spots along the customer journey that customers do not yet recognize.

Considering “The Impact of a Touchpoint on Sales”, the case study could demonstrate that using a statistical analysis together with a customer survey based on previously identified brand-owned touchpoints the digital retailer was able to determine the impact of the brand-owned touchpoints on the sales value of a customer. Hereby our study exemplified that in contrast to previous attribution models (Li and Kannan 2014; Xu et al. 2014), which focused on online customer journeys, our framework was able to successfully (BF10 = 10,739) attribute the sales value generated by each brand-owned touchpoint across online and offline touchpoints.

Generally speaking, the case study could provide the first demonstration that combining a touchpoint identification workshop, employee and customer surveys, sales data, and the statistical instrument of a Bayesian multiple regression, as done in the proposed framework, offers possible solutions to some of the problems identified by Lemon and Verhoef (2016) from a digital retail perspective. As demonstrated in the case study, aggregating these possible solutions, the digital retailer was able to derive a specific marketing action toolset, which was used to influence brand-owned touchpoints with a high probability to impact sales. We argue that influencing these specific touchpoints allows digital retailers to effectively increase their overall sales value and thus increase their conversion rate.

Although our case study tested the proposed framework with one Austrian sports appeal and equipment retailer only, we see no reason why the steps of identifying touchpoints, determining internal and external touchpoint perception, determining the monetary value of touchpoints, identifying sales influencing touchpoints, and deriving appropriate marketing actions are not transferable to retailers from other branches which focus on selling products or services.

## Conclusion

In accordance with the overall goal of the paper (“Offer possible solutions to the set-out issues of digital retail and to use these solutions as building blocks in a conversion rate optimization framework”), we designed a conversion rate optimization framework based on possible solutions for common issues digital retailers face and exemplified its usage with the Austrian subsidiary of an international sports appeal and equipment retailer. Reflecting on the performed case study, the retailer applying the framework could identify probable sales influencing brand-owned touchpoints and derive a specific marketing action toolset to influence these touchpoints. Thus, the framework can be used by digital retailers to increase their competitive power against e-commerce.

To gain this competitive edge, the following recommendations can be given to practitioners, especially from the field of marketing analytics. First, for digital retailers, it is crucial to identify most, if not all, of their brand-owned touchpoints as these are the only customer contact points that can be influenced by the digital retailers themselves making their monetary impact worth analyzing. Second, digital retailers should align the internal and external perception of the identified brand-owned touchpoints to ensure that only touchpoints which are recognized by customers and are also part of the digital retailers’ customer journey management strategy are being analyzed, as this allows for an optimal allocation of company resources and thus goal fulfilment. Third, when analyzing the impact of touchpoints on sales, digital retailers should adhere to advanced statistical methods as the interaction between multiple touchpoint encounters is complex. Thus, simple regression approaches might not lead to accurate predictions of monetary value and should be replaced by their Bayesian counterparts or even more capable machine learning models. However, digital retailers should always ensure that the statistically calculated impact of touchpoints on sales is always viewed in context of other company specific conditions which might have an impact on the specific values calculated. As such, the decision if and why a touchpoint influences sales should be made on the basis of the combination of a company, customer and statistical perspective.

As in every research, our research suffers from limitations that might be avenues for future research in the field of marketing analytics. As such, it must be noted that currently all data used to derive the final marketing action toolset are collected and analyzed manually via surveys, workshops with focus groups, and statistical tools, all relying on a snapshot of the current situation of a digital retailer. Therefore, a significant improvement of the framework’s design would be to use precise tracking tools and data evaluation supported by machine learning techniques in the data collection process. Both would increase accuracy and topicality when identifying the most sales influencing touchpoints of a digital retailer. Additionally, if the process of tracking and evaluating the collected data over time could be eased, or even automated, the framework could increase its practical applicability as it would allow digital retailers to constantly identify their most important customer touchpoints together with possible marketing actions to influence them.

To conclude, as the retail sector moves forward and most retailers face challenges to remain competitive, this research can serve as a foundation for designing conversion rate optimization frameworks to increase the competitive power of digital retailers compared to e-commerce. It will be rewarding to see what insights future research will reveal.
